# Recent Advances on Biomass-Derived Carbon Materials-Based Electrochemical Sensors

**DOI:** 10.3390/molecules30143046

**Published:** 2025-07-21

**Authors:** Dacheng Wang, Yan Deng, Xiaowei Liu, Baoli Wang, Feng Yang

**Affiliations:** 1 Haikou Key Laboratory of Marine Contaminants Monitoring Innovation and Application, Haikou Marine Geological Survey Center, China Geological Survey, Haikou 571127, China; 2 State Environmental Protection Key Laboratory of Monitoring for Heavy Metal Pollutants, Changsha 410019, China; 3 Key Laboratory of Laser Technology and Optoelectronic Functional Materials of Hainan Province, Haikou 571158, China

**Keywords:** biomass-derived carbon, BDCM, electrochemical sensor, environmental pollutants, drugs, biomolecules

## Abstract

Biomass-derived carbon materials (BDCMs) have garnered numerous research interests due to their conspicuous electrochemical merits, which makes them promising candidates for electrode modification materials in electrochemical sensors. This review focuses on the recent progress in BDCM-based electrochemical sensors. We summarize the main synthesis methods and properties of BDCMs and their electrochemical sensing applications in the detection of environmental pollutants, drugs, and biomolecules. This review also emphasizes the advantages and disadvantages of each preparation method, as well as the limitations in detecting the target substance. Furthermore, this review discusses the current challenges and future prospects for advancing biomass-derived carbon materials-based electrochemical sensors.

## 1. Introduction

The electrochemical sensor is the device that performs qualitative or quantitative detection and analysis by measuring the electrochemical signals of target analytes [1,2,3,4,5]. Compared to traditional analytical methods, such as high-performance liquid chromatography, inductively coupled plasma mass spectrometry, and ultraviolet spectroscopy, electrochemical sensing technology has become a research hotspot in analytical chemistry due to its advantages of being inexpensive, highly sensitive, highly selective, and having a fast reaction speed [6,7,8,9]. The basic principle is to use the physical and chemical interactions between the target analytes and the electrode surface to cause changes in the electrochemical signal, which is then converted into visualized electrochemical spectra by an electrochemical workstation, allowing for the qualitative or quantitative detection of target molecules through spectral analysis [10]. Commonly used methods for collecting electrochemical spectra include cyclic voltammetry (CV) [11,12], differential pulse voltammetry (DPV) [13,14,15], linear scan voltammetry (LSV) [16,17], square wave anodic stripping voltammetry (SWASV) [18,19], electrochemical impedance spectroscopy (EIS) [20,21], and the time-ampere method (i–t) [22,23,24]. During the analysis process, the specific capture and recognition of target molecules on the working electrode surface is a critical factor affecting the performance of electrochemical sensors [25,26,27]. Unmodified working electrodes have a narrow detection range, low detection limits, and poor sensitivity. Therefore, effectively modifying the electrode-sensitive materials on the electrode surface to amplify the electrochemical signal is the key to improving the analytical performance of electrochemical sensors.

In recent years, carbon materials have attracted extensive research interest due to their unique structure, broad applicability, high sustainability, economic compatibility, and renewability [28,29,30,31]. In particular, biomass-derived carbon materials (BDCMs) have become a research hotspot of the carbon family. Their applications primarily include catalysis [32,33,34,35], CO_2_ capture [36,37,38,39,40], adsorption separation [41,42,43,44,45], microwave absorption [46,47,48,49], water treatment [50,51,52,53,54], energy storage [55,56,57,58,59,60,61,62], and electrochemical sensing [63,64,65,66,67,68,69]. Biomass-derived carbon materials prepared through rational design and manipulation exhibit regular surface morphology, high conductivity, high porosity, chemical stability, and surface functionalization, making them significant for both research and practical applications. Using BDCMs in electrochemical sensing not only enables the construction of rapid, sensitive, and stable sensors, but it also facilitates waste reuse. In recent years, researchers have focused on enhancing the electrochemical sensing performance of biomass-derived carbon materials through methods such as activation, heteroatom doping, and the loading of metal/metal alloy/metal oxide nanoparticles [70,71]. These methods increase reactive sites and surface defects, enhance the material’s electroactive area, improve material transport capabilities, and boost conductivity, thereby optimizing detection performance.

This article primarily summarizes the preparation technologies of biomass carbon, reviews several common biomass carbon structures, and describes the effects of different preparation methods on the morphology, structure, and electrochemical properties of biomass carbon materials. Additionally, different preparation methods are compared and analyzed in terms of cost, efficiency, and environmental impact. Further, it provides a general summary of its applications as electrochemical sensor electrodes for the sensitive detection of environmental pollutants, drugs, and biomolecules. The performance parameters of electrochemical sensors, including the linear range, sensitivity, detection limit, response time, stability, and reproducibility were compared and analyzed. Future research should focus on further optimizing their structure and performance and exploring new preparation processes and application scenarios to promote the development of more efficient and environmentally friendly sensor technologies.

## 2. Preparation of Biomass-Derived Materials

The synthesis methods of BDCMs vary depending on the type of biomass and its application fields, primarily including pyrolysis, hydrothermal, and molten salt carbonization [72]. The synthesis methods and experimental parameters, such as temperature, pressure, carbonization time, gas atmosphere, solvent, and substrate concentration, play crucial roles in determining the structure and activity of the resulting materials [73]. Rational design and reasonable regulation of these synthesis parameters is essential for tailoring the properties of carbon materials to meet specific requirements.

### 2.1. Pyrolysis Carbonization Method

Most BDFMs are prepared through the high-temperature pyrolysis method in an inert atmosphere such as N_2_ or Ar. During the procedure, the biomass undergoes different chemical pathways, where it first volatilizes water at 100–300 °C and is followed by pre-carbonization at about 400–600 °C, producing gases like CO_2_, CH_4_, H_2_, H_2_O, and CH_4_. Finally, it transforms into a carbon framework at 700 °C or higher, as decarboxylation and dehydration reactions are more favorable at higher temperatures [74]. The pyrolysis method is currently the most widely used method as the structure and properties of the synthesized materials can be controlled by adjusting the experimental parameters [75]. The pyrolysis temperature and heating rate are the critical factors, which influence the biomass pyrolysis reactions and, thus, the product distribution [76].

**Figure 1:**
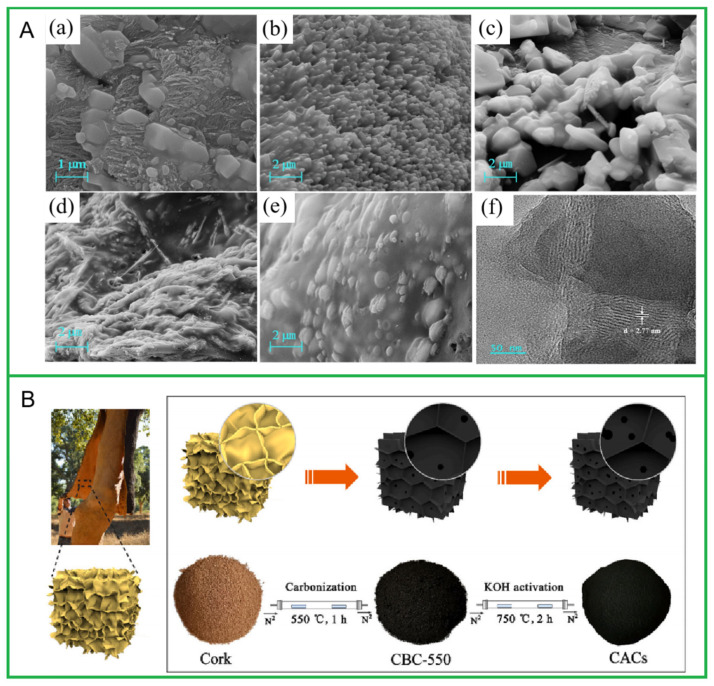
(**A**) SEM and TEM images of the carbon-based materials derived from crab gills. SEM images of CGC-700 (**a**), CGC-800 (**b**), CGC-900 (**c**), CGC-800/GA (**d**), CGC-800/GA/GOx (**e**); TEM image of CGC-800 (**f**). Reprinted with permission from Ref. [77]. (**B**) A flow chart of cork-derived carbon prepared by pyrolysis. Reprinted with permission from Ref. [81].

When preparing carbon materials using pyrolysis methods, biomass can be simply processed without introducing other reagents for direct carbonization. The graphitization degree of carbon materials prepared through direct carbonization is generally low, and it gradually increases when the carbonization temperature exceeds 900 °C. The morphology and pore structure of the prepared materials are also primarily influenced by the gases produced during the carbonization process. For example, Yin et al. [77] collected crab gills, which were crushed and dried as pre-treatments and then pre-carbonized at 400 °C for 1 h, followed by carbonization at 700 °C, 800 °C, and 900 °C for 1 h under a N_2_ atmosphere, resulting in the activated carbon materials CGC-700, CGC-800, and CGC-900 with different structures. Figure 1A shows the SEM and TEM images of the materials, from which it can be seen that the carbon pyrolyzed at 800 °C exhibited a uniform nanorod structure. Meanwhile, at 900 °C, the carbon seemed to aggregate together to form a large blocky structure, which meant the collapse of the carbon frame. The BET results show that CGC-900 had the highest graphitization degree, while CGC-800 had the largest specific surface area (1144.8 m^2^/g). Introducing other catalysts and activators into biomass can effectively improve the graphitization degree and pore structure of the prepared carbon materials. In addition to the in situ introduction of catalysts or activators during the preparation process to modify carbon materials, physical or chemical post-activation treatments can also be used to improve performance. Generally, substances such as KOH, ZnCl_2_, and K_2_CO_3_ are used for activation to further improve the pore structure and specific surface area of the material, resulting in porous carbon. For example, Chang et al. [78] used silk as a raw material, conducted a degumming treatment, and then introduced FeCl_3_ and ZnCl_2_ as catalysts and activators. The resulting silk fibroin was heated to 220 °C under Ar gas for 45 min and then heated to 900 °C for 2 h. After natural cooling, silk-derived carbon was obtained. X-ray diffraction analysis confirmed that the prepared material has high graphitization. FeCl_3_ catalyzes the transformation from amorphous carbon to graphitized carbon, while ZnCl_2_ is first reduced to metallic Zn. As the temperature continues to rise, Zn volatilizes and forms pore structures on the carbon matrix, increasing the specific surface area of the material. Jin et al. [79] collected Azalea Petal to synthesis BDCMs. After washing and drying, the raw materials were ground into powder, soaked in a KOH solution for 3 h, and then carbonized at 800 °C under a N_2_ atmosphere for 3 h to obtain porous carbon materials with a specific surface area of 788.9 m^2^/g. In contrast, the specific surface area of the carbon material obtained without adding KOH during the preparation process was 417.4 m^2^/g. It is evident that the introduction of KOH is an effective method to increase the specific surface area of carbon materials. The steps involved in pyrolysis and carbonization are very complex, but their mechanisms remain unclear due to the complexity of reaction conditions. According to previous studies, BDCMs prepared through pyrolysis at lower temperatures (400~600 °C) has a rich surface with functional groups, making it more suitable for pollutant adsorption. For instance, bamboo BDCMs have been successfully prepared by the pyrolysis method at 500 °C and used for the adsorption of levofloxacin (CFX) and lead ion (Pb^2+^) [80]. The maximum adsorption efficiencies of binary-mixture CFX and Pb^2+^ on bamboo BDCMs reached 99.9 and 90.8%, respectively, while the very-high adsorption capacities attained by the Langmuir model were found to be 183.6 and 285.7 mg/g for Pb^2+^ and CFX, respectively. The mechanisms of single CFX, Pb^2+^, and CFX-Pb^2+^ adsorption are governed by not only electrostatic interactions, but also hydrogen bonding. Similarly, cork-activated carbons (CACs) with a fluffy honeycomb-like structure were synthesized by pyrolysis at 550 °C (Figure 1B). The adsorption performance results show that the CACs have an outstanding maximum methylene blue adsorption capacity (1103.68 mg/g) and fast adsorption kinetics (800 mg/L, 99.8% in 10 min) [81]. Sun et al. [82] pyrolyzed rice straw at 450 °C for 2 h to obtain carbon materials, which exhibited abundant carboxyl groups, lactone groups, and phenolic hydroxyl groups, showing good application prospects in slowing water infiltration in clay soils. Huang et al. [83] used wheat straw, corn stalks, and rice straw as raw materials, pyrolyzed them at 550 °C for 2 h to obtain different carbon materials, and then systematically investigated their effects on the water retention in acidic soils.

The BDCMs prepared by carbonization at temperatures above 700 °C have a certain degree of graphitization and a larger specific surface area, making them more suitable for electrochemical applications. The main drawbacks of pyrolysis include slow reaction rates, a longer preparation process, and the release of gases that can pollute the environment, necessitating the use of appropriate gas absorption devices to treat the gases [84,85].

### 2.2. Hydrothermal Carbonization

**Figure 2:**
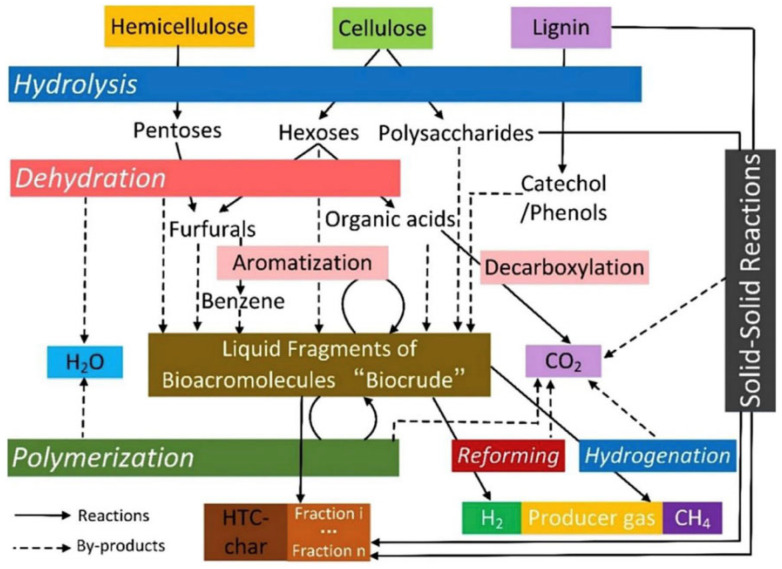
Principle reaction pathways in the HTC process. Reprinted with permission from Ref. [88].

The first experiments of the scientific age were presumably carried out by Bergius, who described, in 1913, the hydrothermal carbonization (HTC) of cellulose into coal-like materials [86], primarily using biomass materials such as plant raw materials, animal waste, agricultural residues, or forestry by-products. Generally, the HTC procedure involves the steps of hydrolysis, dehydration, decarboxylation, condensation/polymerization, and the aromatization process. It is usually carried out at low temperatures, between 100 and 260 °C (typically not exceeding 300 °C), during which the biomass is heated by submerging it in a closed system. Typically, the reaction pressure in the system is generated spontaneously and is associated with the vapor pressure of water in the subcritical state at the reaction temperature [87]. Different types of biomass prominently affect the morphology of the final products. Biomass with high lignin content generally yields larger-sized products. Nevertheless, biomass rich in hemicellulose is more likely to generate smaller particles. Therefore, the selection of raw materials and the control of reaction conditions are critical in the HTC procedure. Figure 2 shows the reaction pathways in the HTC process [88].

Yang et al. [89] synthesized amino-modified carbon quantum dots (CQDs-NH_2_) in one step by the hydrothermal method at 220 °C for 24 h using banana peel as the biomass carbon source and ethylenediamine as the dopant. The results showed that the addition of curcumin had a quenching effect on the blue fluorescence of CQDs-NH_2_. A good linear relationship was observed with curcumin in the concentration range of 1~15 μmol/L with a detection limit of 18.56 nmol/L. Orange peel was effectively transformed into valuable fluorescent CQDs by solvothermal treatment at 200 °C for 10 h with a size of 2~4 nm [90]. Chen et al. [91] dried and ground corn cobs, soaked them in deionized water for 30 min, and then reacted them at 200 °C in a hydrothermal reactor for 6 h. The resulting dark brown suspension was centrifuged, filtered, and dried to obtain CQD powder. Using the same method, CQDs were also obtained from sunflower seed shells and grape seeds. The authors systematically studied the spectroscopic characteristics of the three types of CQDs and used corn cob CQDs as the research object. They developed a fluorescence sensing platform using their peroxidase activity and fluorescence properties to quantitatively detect the biomolecule dopamine. When preparing BDCMs by hydrothermal methods, the solvent can be either pure water or other solvents, and other activators or catalysts can be introduced into the reaction system. For example, Silva et al. [92] used bagasse as the raw material and added phosphoric acid solution as an auxiliary reagent to prepare hydrothermal carbon by a relatively low-temperature (150 °C) hydrothermal reaction for 14 h. The prepared carbon material was in the form of lumps with a rich surface of hydroxyl groups. Nitrogen-doped CQDs were synthesized by sheep manure with glycine through the HTC method. The results showed that the nitrogen-doped CQDs were synthesized when sheep manure was mixed with glycine in a mass ratio of 4:1 and were reacted at 240 °C for 12 h with a quantum yield of 10.63% [93]. In comparison to CQDs that had not been doped with glycine, the nitrogen of N-CQDs increased from 4.21% to 8.08%.

The advantages of the hydrothermal method lie in the low toxicity of reagents used, the ability to adjust the morphology of carbon materials by changing temperature, the use of wet biomass to eliminate drying costs, and the simplicity of the instruments involved. One major drawback is that the hydrothermal reaction takes place in a sealed high-pressure reactor, which can easily lead to safety accidents if not handled properly. Additionally, the final products of the hydrothermal carbonization method are quantum dots or large-sized carbon particles, which are not the optimal choice for sensing electrode materials and are more commonly used in luminescence and catalysis fields [93,94,95].

### 2.3. Molten Salt Carbonization Method

Molten salt carbonization is a method that converts biomass into carbon materials in molten salt media. In the processes, biomass is subjected to high temperatures in the presence of molten salts, resulting in carbonization and activation at lower temperatures than traditional methods. This synthetic method not only reduces production costs, but also simplifies the procedure. Molten salts guarantee uniform and high temperatures, promoting effective thermal treatment and improving reaction kinetics [96]. The resulting products exhibit good thermal stability and excellent heat transfer properties. Most inorganic salts are ionic crystals at room temperature but transform into corresponding ionic liquids or molten salts at temperatures above their melting points. Commonly used molten salts include halides, nitrates, silicates, carbonates, hydroxides, and phosphates of alkali and alkaline earth metal cations. Molten salts have advantages, such as a wide operating temperature range, good thermal stability, low vapor pressure, high heat capacity, low viscosity, and good solubility for many impurities, making them an excellent reaction medium for biomass carbonization and activation [97,98]. They can serve as pore-forming agents during the preparation process and also enable the doping of BDCMs.

**Figure 3:**
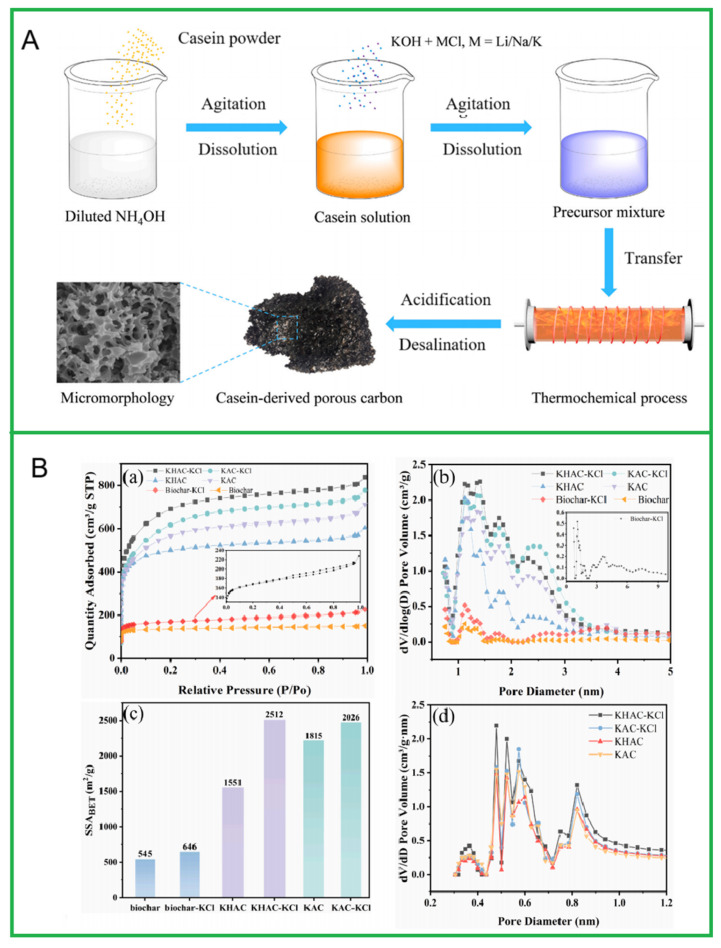
(**A**) Schematic diagram of heteroatom-doped porous carbons synthesized by using casein as a precursor via a thermochemical process of molten salt in conjunction with caustic potash. Reprinted with permission from Ref. [101]. (**B**) (**a**) Nitrogen adsorption–desorption isotherms, (**b**) Pore size distribution curves and (**c**) Specific surface area of KHAC-KCl, KAC-KCl, KHAC, KAC, Biochar-KCl and Biochar; (**d**) Pore size distribution curve tested by carbon dioxide adsorption–desorption. Reprinted with permission from Ref. [102].

The molten salt carbonization method is similar to the pyrolysis carbonization method in that both processes involve the simultaneous carbonization and activation of biomass in molten salt, significantly reducing energy consumption. The main difference lies in using molten salt as the heat transfer medium, which allows for simultaneous carbonization, activation, and impurity removal. During the molten salt carbonization process, the heat released from the biomass can be quickly absorbed, transferred, and stored by the molten salt, making the process self-heating to some extent. Molten salt accelerates the conversion of biomass into BDCMs. Liu et al. [99] synthesized large-pore, highly hydrophobic porous carbon using glucose as the raw material with the assistance of molten salt (LiCl). Li et al. [100] prepared carbon materials by pyrolyzing rice husks mixed with LiCl at 600~800 °C, and they studied the physical and chemical properties of BDCMs using various characterization techniques. Under conditions of 25 °C and 100 kPa, they tested the effects of pyrolysis temperature and the salt/biomass ratio on the structure and CO_2_ adsorption performance of the BDCMs. The results showed that the CO_2_ absorption capacity of the BDCM first increased, then decreased with increasing pyrolysis temperature, and then increased again with higher ratios of molten salt to biomass. In addition to using a single type of molten salt for synthesis, multiple types of molten salts can also be used simultaneously to synthesize BDCMs. Jia et al. [101] employed casein as the precursor, and metal chloride and caustic potash were used together to prepare heteroatom-doped porous carbons via a molten salt thermochemical activation process, as shown in Figure 3. The final product was obtained by soaking, in hydrochloric acid to remove chlorides, carbonates and unreacted KOH. Based on SEM images and N_2_ adsorption isotherms, it has been found that there are different pore-forming mechanisms between pure KOH and the combination of KOH and molten salt, resulting in significant differences in the specific surface area of the final materials. Cheng et al. [102] used KHCO_3_-KCl and K_2_CO_3_-KCl molten salt systems to prepare porous carbon based on corn stalks, and they investigated the effects of the molten salt system on the physicochemical properties of the materials using a series of characterization methods. The results show that both molten salt systems significantly increased the content of oxygen-containing functional groups in the carbon material. However, the KHCO_3_-KCl system was able to significantly increase the proportion of ultra-micro pores (48.6~66.2%) and specific surface area (1551~2512 m^2^/g), while the K_2_CO_3_-KCl system could not. Mechanism studies have indicated that the KHCO_3_-KCl system can significantly influence the interactions between the three components of lignocellulosic pyrolysis reactions, thereby regulating the physicochemical properties of the material and achieving synergistic activation.

When preparing BDCMs using the molten salt carbonization method, different types of molten salts (or combinations of different molten salts, or the combination of molten salts with alkalis) can be used to produce porous carbon-based materials with large specific surface areas. These carbon materials generally exhibit excellent adsorption properties, making their application primarily focused on the field of pollutant adsorption. The main issue currently facing the molten salt carbonization method is how to handle the salts used in the synthesis process. After the reaction, the resulting material is a mixture of carbon and molten salt. When processing the product, filtration and washing are typically employed to remove the molten salt, leaving a large amount of molten salt waste liquid, which is difficult to recycle and reuse. Additionally, the corrosion problem of the reaction vessel is also challenging to address [103,104,105].

### 2.4. Other Carbonization Methods

The microwave-assisted method makes the dipole molecules inside the material move back and forth at a high frequency, and the collision between molecules causes uniform heating inside and outside the material, which improves the carbonization effect. Microwave-assisted carbonization has the advantages of high efficiency, uniform heating, and low cost. Foo et al. [106] reported obtaining a large specific surface area and a porous-structured biomass carbon using the microwave radiation of Mangosteen peel. The specific surface area of biomass carbon was thus determined to be 1098.75 m^2^ g^−1^. Liu et al. [107] used microwave radiation to heat bamboo-based activated carbon, the microporous diameter of which was increased, improving the capability of adsorption. Moreover, a combination of the above carbonization methods is sometimes used in practical situations. Hoang et al. [108] utilized this technique to prepare activated carbon from lignocellulosic biomass, resulting in an enhanced surface area and improved performance in removing heavy metals like Cr, Pb, and Cu. In contrast, Fu et al. [109] employed oxygen-limiting pyrolysis and alkali activation (KOH/NaOH) to produce BDCMs from catkin fiber, achieving a high removal capacity of 82.68 mg/g for Cr(VI). These studies demonstrate the growing importance of optimizing activation methods to maximize the adsorption potential of biomass-based carbons. Their BDCM was synthesized through the microwave pyrolysis of shrimp shell waste (SSW) at varying microwave powers, exploring the yield and properties of the BDCMs in treating landfill wastewater [110]. Their study revealed that the carbon obtained at 900 W was more effective, achieving removal efficiencies of 177~210 mg/g for chemical oxygen demand, 15–64 mg/g for biochemical oxygen demand, and 4.5–8.3 mg/g for phosphate in wastewater. Dong et al. [111] investigated the effects of microwave pyrolysis power and phosphoric acid concentration on the physicochemical properties of modified BDCMs. The results revealed that the most active sample was MHP500–1500. It was prepared at a microwave power of 1500 W with 10 mol/L phosphoric acid at 500 °C. This sample had abundant active sites and excellent electron transfer capability with a specific surface area of 1508 m^2^/g. Table 1 provide the detailed comparisons of the parameters of carbon materials that were prepared by different methods.

Overall, the BDCMs that have undergone high-temperature carbonization (usually above 600 °C) have a higher degree of graphitization and a larger specific surface area. They have better conductivity and can provide more reactive sites, which may lead to excellent detection performance. The main advantage of carbon material prepared by molten salt carbonization is that it has an extremely large specific surface area, which may also provide more active reaction sites for electrochemical reactions. It implies that BDCMs prepared through high-temperature carbonization and molten salt carbonization may be more conducive to the construction of high-performance electrochemical sensors. The surface of BDCMs prepared by the HTC method presents abundant functional groups, which can be used for the adsorption and water purification. However, the resulting carbon usually emerges with a low carbonization degree, porosity, and specific surface area. Therefore, to meet the requirements of high conductivity and the high specific surface area of electrode materials in applications such as electrochemical energy storage and electrochemical sensors, activators need to be added during the HTC process or the HTC carbon products must be modified for post-treatment. In addition, carbon synthesized by HTC results in mostly carbon quantum dots, which presents a relatively low yield and exists in a liquid form. Compared to bulk carbon, the concentration is difficult to control, and the intrinsic properties are not very suitable for electrochemical applications.

## 3. Application of BDCM-Based Materials in Electrochemical Sensors

By designing carbon-based materials with different structures and sizes, and modifying them on the electrode surface, novel electrochemical sensors with unique properties can be prepared [112,113]. The following section mainly summarizes the application and research progress of BDCM-based electrochemical sensors in the analysis of environmental pollutants, drugs, and biomolecules.

### 3.1. Application in Environmental Pollutant Analysis

The transformation of human lifestyles, industrial expansion, and excessive urbanization has made environmental pollution a worldwide problem. Therefore, the application of BDCMs in the electrochemical detection of environmental pollutants is increasingly extensive, and they are often used to detect organic compounds and heavy metals and other environmental pollutants.

#### 3.1.1. Application in Organic Pollutant Analysis

Organic pollutants are highly toxic, persistent, bioaccumulative, and have long-range transport properties. These characteristics make them resistant to degradation in the environment and allow them to accumulate through the food chain, ultimately posing greater harm to humans. Traditional methods for detecting organic pollutants include spectrophotometry (such as Fourier transform infrared spectroscopy (FTIR), Raman spectroscopy (Raman), UV-visible spectrometry (UV-vis), and nuclear magnetic resonance spectroscopy) and chromatography (such as gas chromatography, liquid chromatography, and ion chromatography), with specific methods depending on the nature of the pollutant and detection requirements. In recent years, electrochemical sensing technology has made significant progress in the analysis of organic pollutants. Literature reports on sensors based on BDCMs primarily target N_2_H_4_, organophosphorus pesticides, and phenolic compounds.

**Figure 4:**
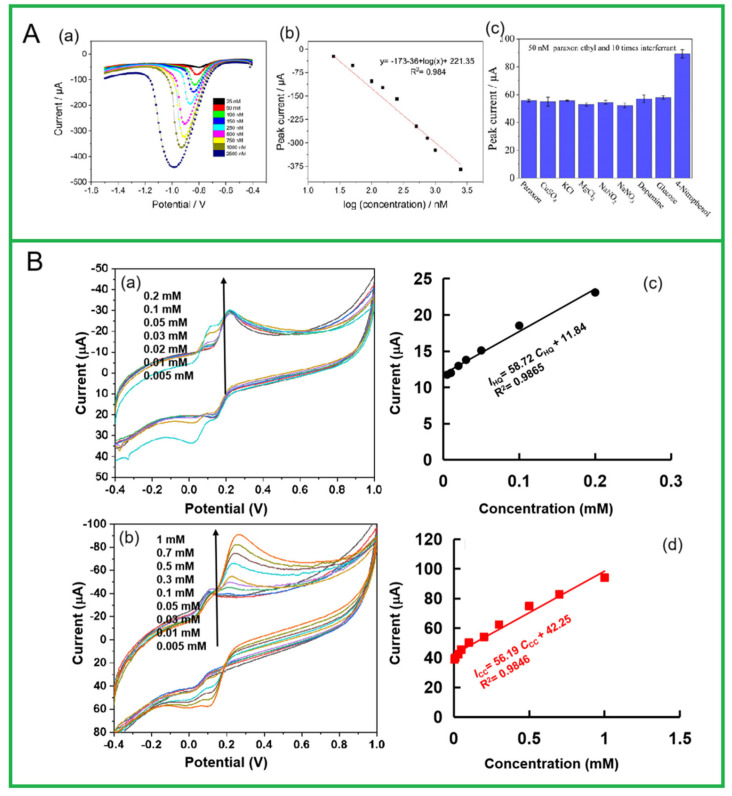
(**A**) Electrochemical detection of paraxon ethyl by SWV from 25 to 2500 nm (**a**), calibration curve with a log of concentration and linear regression equation (**b**), and a depiction of the selectivity investigations of the electrode towards paraxon ethyl (50 nmol/L) in the presence of ten times the interferent concentration (**c**). Reprinted with permission from Ref. [115]. (**B**) CV curves of CLC/GCE in HQ/CC mixtures in 0.1 M PBS (pH 7.0): (**a**) 5~200 μmol/L HQ+100 μmol/L CC; (**b**) 100 μmol/LHQ+0~1 mmol/L CC. The corresponding calibration curves for HQ (**c**) and CC (**d**) of CLC/GCE. Reprinted with permission from Ref. [118].

Sha et al. [114] developed a novel rapeseed-derived carbon quantum dot sensor for detecting N_2_H_4_. The sensor has a sensitivity of 151.5 mA/(mmol·cm^2^) (R^2^ = 0.997) with a detection limit as low as 39.7 μmol/L. The concentration of hydrazine in actual samples, including drinking water and simulated wastewater, was measured using the spiked recovery method with a recovery rate of 97% to 102%, demonstrating good practical application prospects. Adiraju et al. [115] proposed a new electrochemical cyclic activation method in buffer solutions to enhance the electrochemical performance of almond shell BDCMs (AS-BioC). This electrochemical activation method enhances the surface functional groups and porosity of AS-BioC, improving electrode conductivity and the electrochemical active surface area. Activated AS-BioC-modified screen-printed electrodes have exhibit enhanced electrochemical performance, with a detection sensitivity of 17.3 μA/nmol, a detection limit of 1.35 nmol/L, and a wide linear range (25 to 2500 nmol/L) for ethyl paraxon (Figure 4). Madhu et al. [116] prepared nitrogen-doped activated carbon using mango leaves as the carbon source, and the obtained carbon was employed to fabricate the modified electrodes for the detection of 4-nitrophenol (4-NP). The electrochemical performances of the 4-NP sensor were assessed by cyclic and linear sweep voltammetries. Due to the presence of oxygen surface functional groups and heteroatoms in biomass-derived carbon with a high surface area, the constructed sensors show excellent electrochemical activity, with a detection range of 1 to 500 μmol/L and a detection limit of 0.16 μmol/L, achieving a sensitivity of 5.810 μA/(μmol⋅cm^2^). Xu et al. [117] developed an environmentally friendly porous nitrogen-doped carbon for the electrochemical sensing detection of bisphenol A using bamboo pith as the carbon source. The unique porous structure can highly enhance the adsorption and accumulation of bisphenol A, while the rich-doped pyridine-like nitrogen can act as active sites and greatly catalyze the oxidation of bisphenol A. The constructed NDC/GCE presented high sensitivity, a low detection limit, and perfect stability.

By reasonably optimizing the structure and composition of BDCMs, it is possible to simultaneously detect various phenolic compounds in water. For example, Zhao et al. [118] used cotton-derived carbon as an electrode-sensitive material to construct an electrochemical sensor for the simultaneous determination of hydroquinone (HQ) and catechol (CC). The sensor has a wide linear range for HQ (0.5~3000 μmol/L) and CC (1~3000 μmol/L), with detection limits of 0.47 μmol/L and 0.4 μmol/L, respectively. Additionally, the prepared sensor exhibits specific selectivity, a long lifetime, and good applicability. Practical detection results from lake water samples indicate that this sensor has promising application prospects. Similarly, Chen et al. [119] prepared four BDCMs from lychee shell with or without phosphoric acid activation under one- or two-stage heating, and they characterized them by scanning electron microscopy, transmission electron microscopy, infrared spectroscopy, and Raman spectroscopy, respectively. The BDCMs prepared by two-stage heating and phosphoric acid activation (LSC-THP) exhibited a high porosity, the best adsorption capacity, the lowest electric resistance, and the largest electrochemically active surface, and they were then applied to modify a glassy carbon electrode (GCE) after mixing with chitosan (CS) to fabricate the sensing electrode LSC-THP/CS/GCE for the simultaneous detection of CC and HQ. The results indicate that LSC-THP/CS/GCE exhibited the best response current signal at a pH of 6.6 with a linear detection range of 10~2000 μmol/L, and a limit of detections of 1.23 and 0.44 μmol/L was achieved for the CC and HQ detections, respectively. It also exhibited a good anti-interference ability and could be applied to the simultaneous detection of CC and HQ in real samples. Mahfoz et al. [120] synthesized BDCMs directly from jujube leaf waste using pyrolysis and ball milling processes, and they also developed a modified glass–carbon electrode for high-sensitivity and the simultaneous detection of phenolic pollutants, particularly 1-naphthol (1-NP) and 2-naphthol (2-NP). This study developed two innovative electrochemical sensors: the first method quantifies 1-NP and 2-NP using direct oxidation current signals, while the second method uses the redox peak current signals related to quinone formation from the target substances. Electrochemical results show that the detection limit for 1-NP using direct electro-oxidation is 14 μmol/L, and for quinone formation, it is 0.64 μmol/L. Similarly, the detection limit for 2-NP using direct electro-oxidation is 11 μmol/L, which decreases to 0.61 μmol/L. These results surpass traditional detection methods. The green pea peels-derived BDCM (GPPB) was synthesized and characterized using several techniques, and cetyltrimethylammonium bromide (CTAB) surfactant was used to modify GPPB-modified carbon paste electrode for 2,4,6-TCP detection. The optimum values of the limit of detection and quantification were found to be 1.24 nmol/L and 3.78 nmol/L, respectively [121].

#### 3.1.2. Application in Heavy Metal Analysis

Trace metal elements, such as manganese, iron, copper, zinc, and selenium, are crucial for the metabolism of organisms, while other heavy metals like lead, cadmium, mercury, arsenic, and antimony pose severe threats to the environment, affecting the human central nervous system, skin, kidneys, bones, and teeth through the food chain [122]. Currently, electrochemical sensing technology is widely used in the analysis of Hg^2+^, Pb^2+^, Cd^2+^, and Cu^2+^. The detection mechanism primarily involves redox reactions between the electrode-sensitive material and the target analyte, leading to changes in the current signal at the electrode, thus achieving quantitative results. Sensors constructed based on different electrode materials exhibit varying detection limits, sensitivities, and stabilities due to their distinct physicochemical properties.

Silva et al. [92] prepared a Pb^2+^ sensor using carbon paper electrodes modified with bagasse charcoal, and they determined Pb^2+^ using differential pulse adsorption anodic stripping voltammetry (DPAdASV). By optimizing key parameters for detecting Pb^2+^ ions and voltammetry techniques, they achieved a linear response range of 0.50 to 7.06 μmol/L, with detection and quantification limits of 55.0 nmol/L and 181.5 nmol/L, respectively. Using the established voltammetry method, they measured the application of Pb^2+^ in tap water and river water samples, achieving a recovery rate of 91.19% to 109.22%. Xu et al. [123] synthesized worm-like nitrogen-doped carbon frameworks (WNCFs) with abundant edge defect sites using winter melon as the raw material and nitrogen sources. They designed a highly sensitive electrochemical sensor using Nafion-WNCFs for the DPAdASV trace determination of heavy metal Pb^2+^, with a linear range of 0.5 to 100 μg/L and a detection limit of 0.2 μg/L. The BDCM modified on the electrode surface provided additional sites for spontaneous analyte interaction, and the Pb^2+^ was first adsorbed onto the electrode surface with Pb^2+^, which then reduces to Pb^0^. When the potential reaches the oxidation potential of the deposited Pb^0^, it lose electrons and is oxidized back to Pb^2+^ and dissolved into the solution. This oxidation process generates an anode current, and the current intensity is directly proportional to the amount of oxidized metal atoms on the electrode surface. This quantity is, in turn, directly proportional to the original concentration of the measured Pb^2+^ in the solution. Valenga et al. [124] pyrolyzed sugarcane bagasse and activated it with HNO_3_ to develop a DPAdASV method for Cu^2+^ determination. The linear kinetic range was 1.0 to 15.0 μmol/L, with a detection limit of 1.09 μmol/L and good interference resistance. The method was also adequate in terms of accuracy and precision, as well as selective against most of the cationic species commonly found in tap water.

Bressi et al. [125] used beer bagasse as raw materials, which were then simply processed and then reacted with water at 240 °C for 9 h to prepare carbon material (CNDs). The sensor constructed using CNDs as the electrode-sensitive material was able to simultaneously detect Hg^2+^, Pb^2+^, Cd^2+^, and Ni^2+^, with the detection limits of 124 ng/L, 551 ng/L, 453 ng/L, and 608 ng/L, respectively. The corresponding sensitivities were 34.1 μA/(nmol·cm^2^), 21.3 μA/(nmol·cm^2^), 32.2 μA/(nmol·cm^2^), and 11.4 μA/(nmol·cm^2^), respectively. The recovery rate of the spiked seawater and wastewater samples was 99.8% to 102.6%. Sharma et al. [126] prepared parthenium weeds-derived carbon using a combination of hydrothermal and high-temperature carbonization methods. They further used this material to construct a Hg^2+^ sensor, which quantitatively detected Hg^2+^ using DPV, with a detection limit of 6.17 μmol/L and a quantification limit and sensitivity of 18.7 μmol/L and 0.4723 μmol/(μA·cm^2^), respectively. The obtained two-dimensional architecture increased the surface area, while the nitrogen and oxygen functional groups acted as an active site for sensing the mercury ions. Ganaie et al. [127] prepared a composite material SnS_2_@BC by modifying SnS_2_ on staminate flowers-derived carbon. Using this material modified on the GCE surface to construct a sensor, the DPV detection results showed that the detection limits for Pb^2+^ and Hg^2+^ were 0.28 mmol/L and 0.55 mmol/L, respectively.

#### 3.1.3. Application in Environmental Microplastic Analysis

The widespread use of plastic and its slow natural degradation process have led to the accumulation of large amounts of discarded plastics. These plastics enter water environments and remain there for hundreds or even thousands of years, where they are broken down by photochemical processes into microplastic particles. In recent years, microplastics have caused significant disturbances in aquatic and terrestrial ecosystems. Consuming these particles can have severe consequences for wildlife, aquatic life, and human health. Common methods for detecting microplastics include pyrolysis-gas chromatography–mass spectrometry (py-GC-MS), the thermogravimetric analysis technique (TGA), FTIR, and Raman. Among these, Raman and FTIR can detect samples at the micron level, while Micro-Raman can detect samples as small as 1 μm. However, the detection procedures for combined techniques are complex, individual sample analysis takes a long time, and they require high standards from operators. Additionally, there is a lack of dedicated microplastic pyrolyzers, and mass spectrometry data interpretation is challenging. These factors all limit the widespread application of combined detection methods.

**Figure 5:**
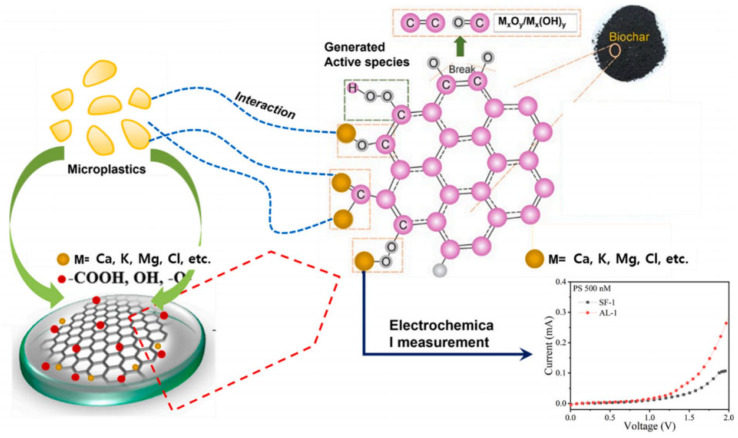
Possible sensing mechanism for the detection of PS over the surface of BDCM-modified GCE. Reprinted with permission from Ref. [129].

Recently, electrochemical sensors have made breakthroughs in the detection of microplastics in the environment: now they are capable of qualitative and quantitative analysis of microplastic particles at the nanometer level within a short period. Nguyen et al. [128] synthesized hydrophobic cerium oxide nanoparticles (CeO_2_ NPs) and used them as electrode modification materials to construct sensors for the first time by employing electrochemical sensing methods to detect two different types of microplastics (polyethylene (PE) and polypropylene (PP)). The CV and LSV electrochemical analysis results showed that the detection limit for PE (27~32 μm) was 0.226 mg/mL and the detection limit for PP (600 μm) was 0.338 mg/mL, with both having a detection range of 0.2~1.0 mg/mL. Additionally, researchers have prepared two types of BDCM-starfish BDCM(SF-1) and aloevera BDCM(AL-1) by naturally carbonizing starfish and aloe vera, respectively, using them as sensor electrode materials for the electrochemical detection of ~100 nm polystyrene microplastics (PS) [129]. Electrochemical results indicate that the sensitivity of the SF-1 electrode is 0.2562 μA/(μmol·cm^2^) with a detection limit of 0.44 nmol/L, while the sensitivity of the AL-1 electrode is 3.263 μA/(μmol·cm^2^) with a detection limit of 0.52 nmol/L. As shown in Figure 5, the difference in the sensing parameters of the two biomass carbon materials may be due to the presence of elements such as Ca, K, and Mg in AL-1 to form functional groups with C, O, and S, and this metal–carbon framework can support PS oxidation, thereby increasing the current.

Although material regulation has enabled the qualitative and quantitative detection of PS down to ~100 nm, the PS detected is merely a theoretical model of microplastics. In reality, microplastics in water are much more complex in both composition and particle size. This is a common issue encountered when using electrochemical sensing technology to detect microplastics. Further research is needed to develop electrochemical sensors that are capable of detecting microplastics in actual water samples.

In the field of pollutant detection, BDCM-based sensors currently cover the detection of various substances, including organic pollutants, heavy metals, and microplastics. Table 2 lists the parameters for environmental pollutant monitoring using different BDCM-based electrochemical sensors, as well as the linear ranges for target analytes with different modified electrodes and detection methods. Overall, the detection limits for environmental pollutants using these sensors have reached the μmol/L or even nmol/L level, with detection ranges mostly ranging from a few μmol/L to several hundred μmol/L. Although some issues in real-world environmental testing have been resolved, further research is needed on the processing of special samples and how to achieve rapid on-site detection. We look forward to future research institutions developing guidelines for the development, data processing, and implementation of pollutant sensors to quickly and accurately detect, analyze, track, and manage contaminated materials [130].

### 3.2. Application in Drug Analysis

In drug testing and analysis, electrochemical sensors based on BDCMs have delivered a great number of research advancements. Overall, the commonly detected targets in chemical drug analysis are acetaminophen and antibiotics. This is mainly because the surface functional groups of the synthesized carbon materials, such as –OH and –COOH, easily interact with these substances physically and chemically, causing significant changes in the electrochemical signal that can be identified, making the detection mechanism relatively clear.

**Figure 6:**
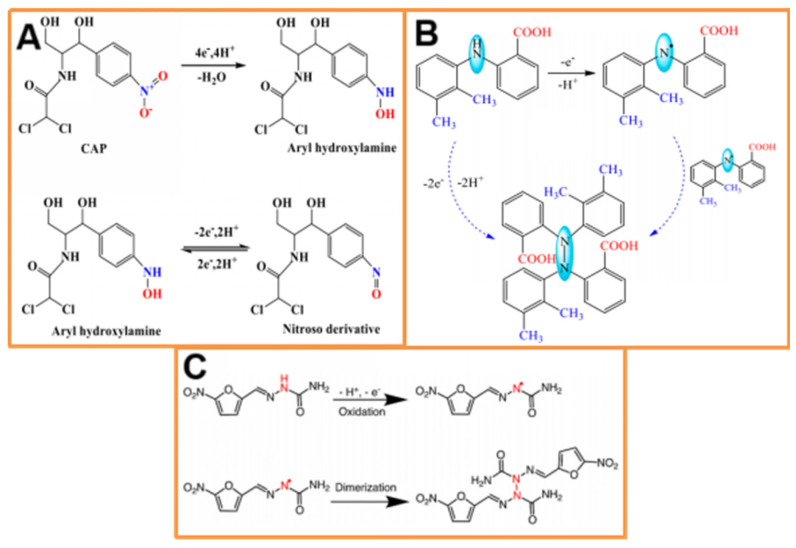
Electro-oxidation mechanism of (**A**) CAP (reprinted with permission from ref. [78]); (**B**) MA (reprinted with permission from ref. [135]); and (**C**) NFZ (reprinted with permission from ref. [136]).

Kim et al. [131] prepared ZnCl_2_-KOH-activated klep-derived carbon to modify GCE for further detection of acetaminophen. The modified electrode showed high sensitivity, selectivity, and a good detection limit for the determination of acetaminophen, with a detection limit of 0.004 μmol/L. Also, the modified electrode showed good results toward acetaminophen even in the presence of ascorbic acid and dopamine, with a detection limit of 0.007 μmol/L. In addition, Prakasam et al. [132] reported the synthesis of phosphorous-doped carbon (PDC) from almond seed skin as a sensing matrix for acetaminophen. The results revealed that the PDC-modified GCE (PDC/GCE) exhibited excellent performance for acetaminophen, with a wide linear range from 0.5 μmol/L to 10 μmol/L. The detection limit (LOD) was calculated to be 0.335 μmol/L. Furthermore, the sensor displayed good stability, reproducibility, and storage stability. N-, S-, and P-doped porous carbon (NSP-PC) derived from elaeagnus gum has been modified on the surface of GCE to fabricate the working electrode NSP-PC/GCE for detecting metronidazole (MNZ), with a detection limit as low as 0.013 μmol/L. The MNZ reduction process has two linear dynamic ranges, from 0.1 to 45 μmol/L and 50 to 350 μmol/L, as well as a low detection limit of 0.013 μmol/L. The constructed sensor has been successfully applied to detect MNZ in pharmaceutical and milk samples with satisfying results. Meanwhile, chloramphenicol, dopamine, nitrophenol, uric acid, glucose, ascorbic acid, vitamin E, and florfenicol have had no interfering effect on MNZ detection [133]. The introduction of heteroatoms can generate more defects in the carbon framework, which act as active sites in the electrochemical process. They can also improve the conductivity of electrodes by acting as electron donors and/or by attracting protons, which facilitates the redox reaction involving nitrogen or a neighboring functional group, thereby affecting the electrochemical properties. Veerakumar et al. [134] used cassia fistula shells as biomass carbon sources, which were carbonized and then loaded with metals to prepare Pt-Re NPs/PAC composites, and they were then used for electrochemical determination of furazolidone, with a detection range of 1 to 299 μmol/L, a detection limit as low as 0.075 nmol/L, and a sensitivity of 5.52 μA/(μmol (cm^2^)). Malode et al. [135] used graphene derived from rice husks to modify carbon paste electrodes for the electrochemical analysis of mefenamic acid (MA) in the presence of CTAB. The constructed modified electrode showed high sensitivity and selectivity for detecting MA with a detection limit of 2.13 nmol/L and a linear range of 0.001–6000 μmol/L. Two electrons were calculated to be involved in the MA mechanism based on the evaluated parameters. Protons and electrons were thus engaged in the electro-oxidation of MA. The electro-oxidation of MA may occur via the approach shown in Figure 6. The sensor showed negligible interference from co-existing molecules, making it suitable for detecting mefenamic acid in human urine, blood serum, breast milk samples, and pharmaceutical tablets with higher recovery.

Liu et al. [136] converted crab shell waste into a porous BDCM at high temperatures to establish an electrochemical sensor for the quantitative detection of nitrofurazone (NFZ). SEM, XRD, FTIR, and N_2_ adsorption–desorption were used to characterize the morphology and structural properties of the crab shell carbon at different temperatures. Electrochemical results showed that the crab shell carbon prepared under 700 °C provided enhanced sensing capability for rapid NFZ detection, with a linear range of 0.40–80 μmol/L, a sensitivity of 0.55 μA/μmol, and a detection limit of 0.11 μmol/L (S/N = 3). This sensor can be used to quantify NFZ in the real drug (compound cod liver oil ointment) with satisfactory recovery. Similarly, Ramadhass et al. [137] reported the preparation of porous activated carbon (3D-PAC) from the biomass waste material, such as the Borassus flabellifer (Asian palmyra palm) shell, as a carbon precursor. They probed the analytical behavior of 3D-PAC-modified GCE to detect FZ. The effect of experimental parameters, like modifier loading concentration/volume, pH, analyte accumulation time, scan rate, and sample concentration, was studied based on the reduction peak current. The developed drug sensor exhibited excellent FZ detection performance, with a wide linear range from 0.5 to 290 μmol/L, a limit of detection (LOD) of 0.5 nmol/L, and appreciable sensitivity of 5.05 μA/(μmol⋅cm^2^). Chang et al. [78] group used the prepared silk carbon composite material as an electrode material to prepare modified electrodes, which were used to detect chloramphenicol (CPA) using DPV technology. The DPV peak current showed a linear relationship with increasing concentration, with a detection range of 1~200 μmol/L and a low detection limit of 0.57 μmol/L (S/N = 3). Based on this, the practicality of the sensor for CPA detection was evaluated using fish, shrimp, and chicken as analytical objects, with a recovery rate of 85.96%~106.72% when using the spiked recovery method and a low RSD.

In addition to its widespread application in chemical drug testing, BDCM-modified electrodes can also be used for the detection and analysis of traditional Chinese medicine molecules. Cheng et al. [138] prepared an Au-Pt@BPC composite by carbonizing oyster mushrooms and then hydrothermally loading Au and Pt nanoparticles onto it, which was modified on the surface of CILE as a working electrode for detecting baicalein. Under optimal conditions, the linear range of this sensor is 0.48~2.0 μmol/L and 4.0~140.0 μmol/L, with a detection limit as low as 0.01 μmol/L. The detailed mechanism of baicalein on the electrodes can be explained by the fact that the reversible process with the first and the second phenolic hydroxyl on the ortho-position of baicalein are oxidized at 0.4~0.45 V and the electrochemical oxidation occurs at the third residue phenolic hydroxyl group [139]. Ai et al. [140] synthesized banana peel-derived porous carbon using high-temperature pyrolysis, and they further combined it with multi-walled carbon nanotubes through self-assembly, which was then modified onto the surface of a GCE to construct an electrochemical sensor for the sensitive determination of baicalein. The electrochemical response of baicalin was studied using CV, and the results showed that the constructed sensor had high sensitivity and a low detection limit.

In addition, the detection mechanism of such substances is currently believed to involve the oxidation of hydroxyl groups in baicalein by electrode materials into carbonyl groups, producing distinct redox peak signals. Literature reports on electrochemical sensors based on BDCMs or other materials have shown that there are relatively more studies on flavonoids in traditional Chinese medicine analysis compared to those focusing on other herbal substances. This may be related to the complexity of the components of the tested substances in traditional Chinese medicine. Current research priorities also tend to focus on reducing the detection limit of these sensors and expanding their detection range. Table 3 summarizes the detection parameters for different drugs using BDCM-based sensors. When detecting chemical drugs, sample processing is relatively simple, making the testing convenient and reliable. However, the actual sample processing for traditional Chinese medicine is slightly more complex, and there are often many structurally similar compounds within the same herb, which requires high selectivity from the sensor or the ability to detect multiple substances simultaneously.

### 3.3. Application in Biomolecular Analysis

Biological molecules are substances with different functions that exist in cells and living organs, mainly including carbohydrates, lipids, proteins, amino acids, vitamins, and nucleic acids. At present, biological carbon-based sensors are mainly focused on the analysis of glucose, dopamine, uric acid, and ascorbic acid, while the detection application of other substances is relatively less.

Li et al. [141] used willow catkins as biomass precursors to prepare bamboo-leaf-shaped CuO nanorods and hollow carbon fiber composites (CuO NRs@PC), which were used to construct non-enzymatic glucose sensors with a wide detection range (5~8000 μmol/L), a detection limit as low as 0.1 μmol/L, and a sensitivity of 609 μA/(mmol⋅cm^2^). The improvement in detection performance may be due to the significant enhancement of mass transfer rates by the three-dimensional porous structure of the carbon matrix, and the good electrical conductivity of the material facilitates electron transfer between biomolecules and the electrode surface. Additionally, defects and micropores in the material provide effective active sites for glucose analysis adsorption. Qu et al. [142] used rose as a carbon source and loaded CoS nanoflowers after carbonization to prepare CoS@C composites, which were modified on the GCE surface to obtain Nafion/CoS@C/GCE working electrodes for glucose detection, with a detection range of 10–960 μmol/L and a detection limit of 2 μmol/L. The main drawbacks of the aforementioned non-enzymatic methods are low selectivity and poor sensitivity. To achieve better selectivity and excellent sensitivity, Shan et al. [143] immobilized glucose oxidase (GOD) on vine-derived 3D porous carbon (3D-CVS) to develop a novel glucose biosensor with a linear response range of 0.58–16 mmol/L and high sensitivity and low detection limits.

Padmapriya et al. [144]. proposed a simple method for the hydrothermal synthesis of phosphorus and nitrogen-doped carbon quantum dots (N, P-CQD) using banana flower bud extract as the raw material. They constructed sensors using N, P-CQD as the modifier to detect dopamine. The doping of heteroatoms increased the conductivity of CQD, and groups such as –COOH, –NH_2_, and –PO_4_^3−^ on the material surface selectively were found to attract cations, leaving anions in the solution due to electrostatic repulsion. Therefore, the constructed sensor had high selectivity and sensitivity for dopamine, with a low detection limit (500 pmol/L) and a wide linear range (6.0~0.1 mmol/L). Additionally, dopamine was successfully detected in real-time samples injected with dopamine, with a detection limit as low as 630 pmol/L and a linear range from 2.5 mol/L to 0.16 mmol/L.

In the analysis of biomolecules, most constructed sensors have not been based on pure BDCMs but rather on non-metallic N and P-doped BDCMs or complexes loaded with metal compounds such as CuO and CoS. This may be due to the special structure of biomolecules, which makes it difficult for them to interact with pure BDCM frameworks; thus, other non-metals or metals must be introduced to promote reactions. Additionally, the indirect detection of biomolecules can be achieved by introducing other substances onto electrodes. For example, Jin et al. [79] used azaleas as a carbon source, prepared hierarchical porous carbon materials (KACMs) through high-temperature calcination and KOH activation, and then assembled KACM and thiocyanate (Thi) simultaneously on the surface of a glassy carbon electrode to construct a Thi/KACM electrochemical sensor for detecting ascorbic acid and uric acid. Electrochemical test results showed that the sensor has strong catalytic activity for the oxidation of ascorbic acid and uric acid, with good separation between the two oxidation peaks. Under optimized conditions, it has a wide linear range (0.05~9 mmol/L) and a low detection limit (ascorbic acid 6.4 μmol/L, uric acid 10 μmol/L). Moreover, the biosensor exhibited good selectivity, stability, and reproducibility. Using Thi/KACM/GCE to simultaneously detect UA in human urine, the recovery rate was 99.4%~101.0%. Valenga proposed an electrochemical sensor based on copper ions (Cu^2+^) immobilized on BDCMs for the indirect determination of the target substance [145]. First, Cu^2+^ is enriched on the surface of carbon paste electrodes modified with BDCMs from bagasse. Then, creatinine is further enriched on the surface, as the formation of creatinine–copper complexes leads to the inhibition of the voltammetric signal of Cu^2+^ (CPE/biochar/copper), which serves as an electrochemical analytical indicator. The linear detection range is 300 to 700 µmol/L, with a detection limit and quantification limit of 91.0 µmol/L and 300 µmol/L, respectively. The standard addition method was used to detect creatinine in the simulated urine samples, with a quantification limit concentration of 300 µmol/L and a recovery rate of 101 ± 7%.

For real-sample testing, such as in blood or urine matrices, there may still be some practical challenges. Both blood and urine samples are mixtures with certain turbidity. Before conducting sample tests, they need to be processed to extract clear liquids, and interference in the samples should be prevented at the same time. In addition, when using human body samples, ethical approval must be submitted. Researchers must be aware that they should obtain consent before using these types of samples in their research. In general, in addition to the analysis of glucose, dopamine, ascorbic acid, and uric acid mentioned above, more detectable biomolecules should be explored in the future. On the basis of improving the sensitivity and other specific indicators of the sensor, the scope of the target substances for detection should be expanded, and the application fields of the sensor should be further broadened.

BDCMs themselves are an excellent base material or electrode material, possessing excellent physical and chemical properties (conductivity, specific surface area, etc.). However, they lack the ability to specifically recognize biomacromolecules, such as antigens, antibodies, and RNA. The main reasons for this are as follows: (i) Although their surface may contain some functional groups, these groups cannot specifically recognize and bind to specific RNA sequences. This demands the introduction of a specific recognition module. (ii) The nonspecific adsorption is abundant, which will seriously interfere with the sensor’s signal (increasing background noise, reducing sensitivity, and causing false positives/false negatives), especially when detecting trace RNA as the problem is particularly prominent then. To address this, researchers have made considerable efforts in the field of constructing sensors with BDCMs for the detection of biomacromolecules. For instance, Velanga et al. [146] produced BDCMs from sugarcane bagasse pyrolysis to immobilize biomolecules in order to assemble an electrochemical immunosensor to detect antibodies against SARS-CoV-2. Under the best set of experimental conditions, negative and positive serum sample responses were distinguished based on a cutoff value of 82.3% at a 95% confidence level. The immunosensor showed selective behavior to antibodies against yellow fever, and its performance was stable to up to 7 days of storage. Sobhan [147] fabricated a novel activated BDCM-based immunosensor for the rapid detection of *E. coli* O157:H7. The gold electrode was coated with the field corn stalk-derived carbon and then functionalized with streptavidin as a linker, which was then further immobilized with biotin-labeled anti-E. coli polyclonal antibodies (pAbs). Binding of anti-*E. coli* pAbs with *E. coli* O157:H7 lead to a significant increase in the electrochemical impedance from 3.5 to 8.5 kΩ when compared to an only biochar-coated electrode. The resulting sensor can be used to detect *E. coli* O157:H7 cells with a LOD of 4 log CFU/mL without incubation.

Overall, appropriately regulating the pore structure, defect, and doping atoms of BDCMs is conducive to the construction of high-performance electrochemical sensors. However, it is possible to introduce some metals in the construction of the sensor, such as SnS_2_@BC [127], CuO NRs@PC [141], Nafion/CoS@C/GCE [142], and CPE/biochar/copper [145], in order to improve the detection performance. The toxicity and leaching risks of metal-containing BDCM-modified electrodes are issues that cannot be ignored in practical applications. The risks mainly stem from the toxicity of the metal itself and the instability of the modified layer in complex usage environments. By carefully selecting low-toxic/inert materials, optimizing the modification process to enhance stability, applying effective protective coatings, strictly controlling electrochemical operation conditions, and conducting rigorous leaching tests and biological safety evaluations, these risks can be significantly reduced, enabling the metal-containing BDCM-modified electrode technology to be safely applied in various sensing fields, especially in the highly safety-demanding biomedical industries.

## 4. Conclusions and Outlook

The detection performance of electrochemical sensors based on BDCMs is primarily influenced by factors such as the composition, structure, surface functional groups, and morphology of the carbon material. Additionally, the detection limit, linear range, and sensitivity of the sensor are related to specific sensor construction parameters, such as the type of electrolyte, the volume of carbon material modified on the electrode surface, the pH of the electrolyte, and ionic strength. Currently, sensors constructed for environmental pollutants, drugs, and biomolecules mainly use traditional large-scale electrochemical workstations as detection systems, with there being relatively less development in portable sensor research. Furthermore, mechanisms for enhancing the performance of constructed sensors and their sensing principles require further in-depth exploration. For the development of BDCM-based electrochemical sensors, future attentions should be focused on the following:

The improvement of electrochemical sensor performance depends on the rational design of modified electrodes, which is mainly related to the intrinsic electrochemical activity of the electrode-sensitive materials and surface modification techniques. In the future, in the synthesis of carbon materials, it should be possible to achieve a clear composition and tunable morphology structure of BDCMs, which will not only help improve detection performance, but also facilitate the exploration of sensing mechanisms. Additionally, using emerging molecular imprinting technology to modify electrodes can enhance their affinity and selectivity. Further research is needed into the combined application of carbon materials and molecular imprinting technology.

Electrochemical sensors show promising development trends in detecting various toxic environmental samples, and BDCM-based electrochemical sensors are gradually evolving into portable devices. The integration of new 3D printing technology and miniaturized electrochemical workstations can lead to the development of low-cost, high-sensitivity portable electrochemical sensors. At present, Zhong et al. [148] and Papavasileiou [149] have employed 3D printing technology to construct sensor electrodes using carbon black and polylactic acid as raw materials for the detection of dopamine and hydroquinone, respectively. The concentration range for detecting dopamine was between 0.01 nmol/L and 30 nmol/L with a low LOD of 0.0094 nmol/L. And the linear range for hydroquinone is 0.03 to 24.2 μmol/L with a LOD value of 0.015 μmol/L. In the future, this technology can be further extended to build intelligent, portable BDCM-based sensors for on-site, rapid, and intelligent analysis and detection.

The analysis of sensing mechanisms and performance enhancement mechanisms is also a critical issue that must be thoroughly explored in the development of BDCM-based electrochemical sensors. In the future, it is necessary to combine experimental results with theoretical calculations to clarify the critical mechanisms, further optimize their performance, and to promote the development of BDCM-based electrochemical sensors.

## Figures and Tables

**Table 1:** Comparison of the parameters of carbon materials prepared by different methods.

Methods	Temperature (°C)	Method Characteristics	Prepared Materials Characteristics	Applications	Refs.
Pyrolysis carbonization	400~600	Simple operation, easy to control, easy industrialized, high energy consumption	Abundant functional groups, low graphitization degree	Adsorption	[77,82,83]
	700~1200		High graphitization degree, high conductivity	Capacitors, electrocatalysis, electrochemical sensing, CO_2_ capture	[78,79]
Hydrothermal carbonization	100~300	Low synthesis temperature, rapid reaction, low energy consumption	Abundant functional groups, low graphitization degree; the morphology is mostly carbon quantum dots or carbon particles.	Fluorescence sensing, electrocatalysis	[89,90,91,92]
Molten salt carbonization	900~1200	High thermal efficiency, uniform temperature distribution, reduced energy consumption, enhanced reaction rates	High graphitization degree, large specific surface area	Capacitors, electrocatalysis, electrochemical sensing, CO_2_ capture	[99,100,101]

**Table 2:** The detection parameters of environmental pollutants by different BDCM-based electrochemical sensor methods.

Modified Electrodes	Detection Method	Analyte	Linear Range (μmol/L)	LOD (μmol/L)	Refs.
CQD/GCE	I-t	N_2_H_4_	/	39.7	[114]
AS-BioC/SPCE	SWV	Ethyl paraxon	0.025~2.5	1.63 × 10^−3^	[115]
AC900/GCE	LSV	4-nitrophenol	1~500	0.16	[116]
NDC/GCE	DPV	Bisphenol A	1.0~50.0	1.068	[117]
CLC/GCE	CV	HQ	0.5~3000	0.47	[118]
		CC	1.0~3000	0.40	
DLSNC/GCE	CV	1-NP	1.0~25	0.64	[120]
		2-NP	1.0~25	0.61	
LSC-THP/CS/GCE	DPV	HQ	10~2000	1.23	[119]
		CC	10~2000	0.44	
HC/CPE	DPAdSV	Pb^2+^	0.50~7.06	0.055	[92]
WNCF/BFGCE	DPAdSV	Pb^2+^	0.5~100 μg/L	0.2 μg/L	[123]
BDCM+CPE	DPAdSV	Cu^2+^	1.0~15.0	1.09	[124]
CNDS/SPCE	SWASV	Hg^2+^ Pb^2+^ Cd^2+^ Ni^2+^	/	124 ng/L 551 ng/L 453 ng/L 608 ng/L	[125]
CGC-600/GCE	DPV	Hg^2+^	10~100	6.17	[126]
SnS_2_@BC/GCE	DPV	Pb^2+^ Hg^2+^	/	0.28 0.55	[127]
AL-1/GCE	I-V	PS (100 nm)	/	520	[129]
SF-1/GCE	I-V	PS (100 nm)	/	440	[129]

**Table 3:** The detection parameters of drugs by electrochemical sensor methods based on different BDCMs.

Modified Electrodes	Detection Method	Analyte	Linear Range (μmol/L)	LOD (μmol/L)	Refs.
PNC/PGE	DPV	Chloramphenicol	1~200	0.57	[78]
ZKAKC/GCE	DPV	Acetaminophen	0.01~20	0.004	[131]
PDC/GCE	DPV	Acetaminophen	0.5~10	0.335	[132]
NSP-PC/GCE	LSV	Metronidazole	0.1~45 50~350	0.013	[133]
Pt—Re NP/PAC/GCE	LSV	Furazolidone	1~299	0.075	[134]
RHG/CPE	SWV	Mefenamic acid	0.001~6000	2.13 × 10^−3^	[135]
C-CS-700/GCE	DPV	Nitrofurazone	0.4~80	0.11	[136]
3D-PAC/GCE	DPV	Furazolidone	0.5~290	0.5 × 10^−3^	[137]
Au-Pt@BPC/CILE	DPV	Baicalein	0.48~2.0 4.0~140.0	0.01	[138]
BPBC-MWCNT/GCE	DPV	Baicalein	0.004~100	1.33 × 10^−3^	[140]

## Data Availability

No new data were created or analyzed in this study. Data sharing is not applicable to this article.
